# Rehabilitative skilled forelimb training enhances axonal remodeling in the corticospinal pathway but not the brainstem-spinal pathways after photothrombotic stroke in the primary motor cortex

**DOI:** 10.1371/journal.pone.0187413

**Published:** 2017-11-02

**Authors:** Naohiko Okabe, Naoyuki Himi, Emi Maruyama-Nakamura, Norito Hayashi, Kazuhiko Narita, Osamu Miyamoto

**Affiliations:** Second Department of Physiology, Kawasaki Medical School, Matsushima, Kurashiki City, Okayama, Japan; Uniformed Services University, UNITED STATES

## Abstract

Task-specific rehabilitative training is commonly used for chronic stroke patients. Axonal remodeling is believed to be one mechanism underlying rehabilitation-induced functional recovery, and significant roles of the corticospinal pathway have previously been demonstrated. Brainstem-spinal pathways, as well as the corticospinal tract, have been suggested to contribute to skilled motor function and functional recovery after brain injury. However, whether axonal remodeling in the brainstem-spinal pathways is a critical component for rehabilitation-induced functional recovery is not known. In this study, rats were subjected to photothrombotic stroke in the caudal forelimb area of the primary motor cortex and received rehabilitative training with a skilled forelimb reaching task for 4 weeks. After completion of the rehabilitative training, the retrograde tracer Fast blue was injected into the contralesional lower cervical spinal cord. Fast blue-positive cells were counted in 32 brain areas located in the cerebral cortex, hypothalamus, midbrain, pons, and medulla oblongata. Rehabilitative training improved motor performance in the skilled forelimb reaching task but not in the cylinder test, ladder walk test, or staircase test, indicating that rehabilitative skilled forelimb training induced task-specific recovery. In the histological analysis, rehabilitative training significantly increased the number of Fast blue-positive neurons in the ipsilesional rostral forelimb area and secondary sensory cortex. However, rehabilitative training did not alter the number of Fast blue-positive neurons in any areas of the brainstem. These results indicate that rehabilitative skilled forelimb training enhances axonal remodeling selectively in the corticospinal pathway, which suggests a critical role of cortical plasticity, rather than brainstem plasticity, in task-specific recovery after subtotal motor cortex destruction.

## Introduction

Stroke is a major cause of disability worldwide and rehabilitation is commonly used to treat chronic stroke patients. Disability of the upper extremities is a common impairment experienced by a large majority of stroke survivors. Many forms of rehabilitative therapy, especially task-specific training, have long been used to improve upper limb dexterity following stroke. In the recent guideline for stroke rehabilitation, task-specific training is still considered to be a more beneficial and reliable form of therapy than newly developed rehabilitative methods such as transcranial magnetic stimulation, transcranial direct current stimulation, robotic therapy, or virtual reality [1]. Furthermore, the majority of recently developed or developing therapies are expected to be combined with task-specific training, implying a central role of task-specific training in future stroke therapies [2]. However, despite its popularity in clinical settings, the precise mechanism by which task-specific training promotes functional recovery remains to be elucidated.

After stroke, surviving neurons undergo morphological alterations such as axonal remodeling, dendritic arborization, and synapse formation to compensate for lost functions. Through these morphological changes, surviving neurons modify their innervations and reorganize remaining neural networks. The functional contribution of the newly formed connections has recently been proven using pathway-specific silencing techniques [3–5]. Because of the significant relevance between cortical plasticity and functional recovery after stroke, most of the studies involving axonal remodeling after brain damage have focused on the cortical pathways such as the corticospinal [3,6,7] and corticorubral pathways [4,5]. In addition to the corticospinal tract, the spinal cord receives neural input from various descending spinal pathways from the deep brain areas, including the rubrospinal tract (from the red nucleus), the reticulospinal tract (from the reticular formation), the vestibulospinal tract (from the vestibular nuclei), and the tectospinal tract (from the superior colliculus). These brainstem-spinal pathways are also known as the extrapyramidal system. Although these pathways have been generally known to be involved in gross motor control [8], a recent study has demonstrated significant involvement of the reticulospinal tract in skilled motor behaviors [9]. Furthermore, Bachmann et al. have demonstrated increased brainstem-spinal projections after cortical stroke in mice [10]. The strengthened input from the reticulospinal pathway was also demonstrated with electrophysiology in macaque monkeys after corticospinal tract lesion [11]. These data suggest the involvement of descending spinal pathways from the deep brain areas in functional recovery after stroke. On the contrary, maladaptive plasticity, or hyperexcitability in the reticulospinal tract, is considered to be a plausible mechanism for post-stroke spasticity, which is a common limitation to recovery in stroke survivors [12]. Therefore, better understanding of the neural plasticity in the brainstem-spinal pathways is necessary for better therapeutic strategies after stroke. However, whether rehabilitative training can promote axonal remodeling in the brainstem-spinal pathways is not known.

The goal of the current study was to investigate the effect of rehabilitative training on axonal remodeling in the brainstem-spinal pathways during rehabilitation-induced functional recovery. We carried out rehabilitative training using a skilled forelimb reaching task (task-specific training) after subtotal destruction of the caudal forelimb area (CFA) in the primary motor cortex and analyzed the rehabilitation-induced alterations in the brainstem-spinal projections using a retrograde tracing method.

## Materials and methods

### Animals

We used a total of 44 adult male Fisher 344 rats (9 weeks old at the beginning of training; 160–180 g; CLEA, Tokyo, Japan) for the experiments. We chose to conduct our experiments in Fisher 344 rats, because neural network remodeling and motor map reorganization induced by rehabilitative training have been well investigated in this strain [13–15]. Each rat was housed in a temperature-controlled vivarium on a 12-hr:12-hr light:dark cycle. To motivate the rats for the pellet reaching task, their food intake was moderately restricted throughout the study, to maintain their body weight at 80% of the *ad libitum* weight. Water was available *ad libitum*. All experimental procedures were in accordance with National Institutes of Health regulations and approved by the Animal Research Committee of Kawasaki Medical School.

### Experimental design

**Fig 1:**
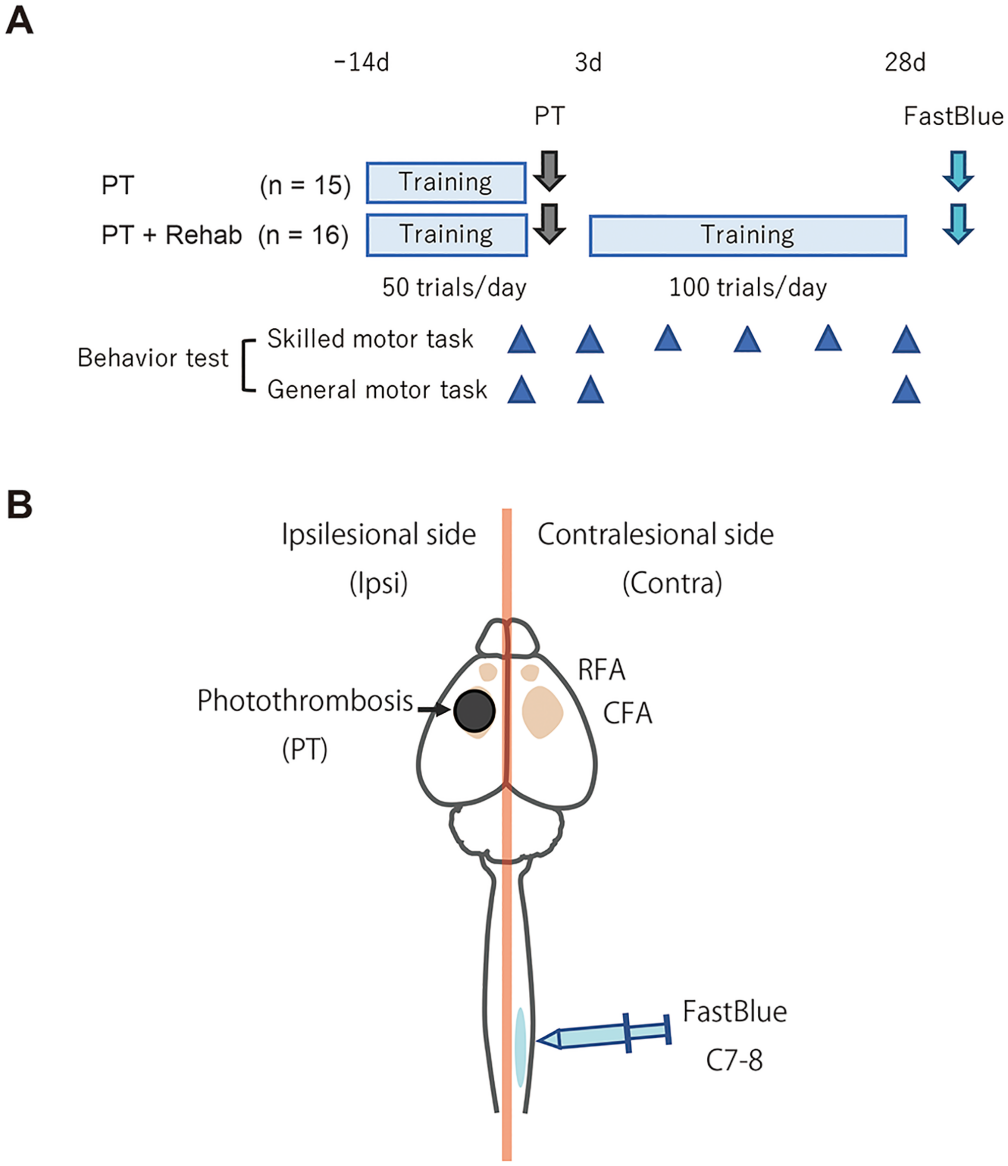
Experimental design. (A) After completion of skill acquisition training, rats received photothrombotic stroke (PT) in the CFA. Rat with rehabilitative training (PT + Rehab) carried out a skilled forelimb reaching task 100 times per day, 5 days per week for 4 weeks. Fast blue was injected 3 to 4 days after final behavioral testing. (B) Fast blue was injected into the lower cervical spinal cord (C7-8) in the contralesional side. Orange areas indicate forelimb areas (divided into RFA and CFA). Black circles indicate infarct induced by photothrombosis.

We investigated the effects of rehabilitative skilled forelimb training (Rehab) on functional recovery and axonal remodeling in the descending spinal pathways after photothrombosis (PT) (Fig 1A). Rats were randomly divided into either PT (n = 15) or PT + Rehab (n = 16). The sample size was chosen on the basis of our pilot experiments and those reported in previous publications [6, 10]. After completion of acquisition training for the skilled forelimb reaching task, rats underwent PT in the CFA. Rehabilitative training was carried out using the skilled forelimb reaching task beginning 4 days after PT and ending 28 days after PT. To evaluate functional recovery after PT, behavioral tests were conducted for both skilled motor tasks (skilled forelimb reaching test and staircase test) and general motor tasks (ladder walk test and cylinder test). Skilled motor performance was evaluated on the day before PT and 3, 7, 14, 21, and 28 days after PT. To avoid the lowering of motivation due to repetitive testing, general motor performance was evaluated only on the day before PT and 3 and 28 days after PT. Three to four days after final behavioral testing, all rats received an injection of Fast blue into the lower cervical spinal cord (C7-8) in the contralesional side (Fig 1B). The rats were number-coded, and both the behavioral assessments and data analysis were carried out by an examiner who was blinded to the experimental groups.

### Behavioral training and testing

#### Skilled forelimb reaching task

The skilled forelimb reaching task was carried out as previously described [15], with a slight modification. For the acquisition of skilled forelimb reaching, rats received training on 5 sequential days per week. The training consisted of 1 week of food tray tasks and 3 weeks of single pellet retrieval tasks. To habituate the use of the forelimbs, rats performed the food tray task for 1 week as previously described [16]. Briefly, a food tray (4 cm long × 7 cm wide × 4 cm deep) that was filled with food every 24 h was mounted in front of the rat’s wire mesh cage. After the food tray task, rats carried out the skilled forelimb reaching task using a Plexiglas reaching box (25 cm high × 10 cm wide × 25 cm long; Kagawakikai, Takamatsu, Japan) that had a tall narrow window (25 cm high × 1 cm wide). A 2 cm wide × 4 cm long shelf was mounted in front of the window on the outside of the wall, 3 cm above the floor. On the first 2 days, the rats were placed in the reaching box, which had food pellets (45 mg; Bioserve, Flemington, NJ, USA) scattered on the cage floor and on the shelf, for 1 h. From the third to the fifth days, the rats were placed in the box for 20 minutes. Single pellets were initially placed within tongue distance, then gradually placed at greater distances until the rats could retrieve a pellet from the shelf 2 cm away from the inside wall. During the first week, this procedure usually enabled the rats to retrieve single pellets using only the preferred forelimb. In the current study, we did not correct for the handedness of the rats, because we did not observe any difference in motor performance between left- and right-handed rats. To prevent rats from rashly reaching and to keep constant training and testing conditions, a pellet was not given when part of the body contacted the front wall. After shaping the reaching behavior, rats performed 50 trials of training per day for 2 weeks, and pre-stroke motor performance was calculated by averaging the results of last 3 days prior to PT. Post-stroke motor performance was evaluated in the 3 days after PT, and weekly thereafter, for 4 weeks. We examined motor performance in a testing session of 50 trials with a maximum time of 10 min. In our previous study [15], this weekly testing session was sufficient to maintain motor performance in sham operated rats. Testing sessions were recorded on video and analyzed by an examiner who was blinded to the experimental groups. We counted the number of trials and first reach successes, and used the first reach success rate (first reach successes/trial) for the motor performance score, because in our previous study, the first reach success rate was the most sensitive measure of motor deficits after stroke. In the final testing session, we also counted the numbers of reaches and total successes (= number of pellets eaten), and calculated the total success rate (total successes/trial) and percentage of successful reaches (total successes/reach). Rats were excluded if the average first reach success rate was lower than 30% in the pre-stroke examination. We excluded PT rats whose post-infarct motor performance rate was greater than 50% in order to conduct experiments using only severely affected rats. All rats that satisfied the inclusion criteria for the skilled forelimb reaching test were included in the study. Rehabilitation training consisted of 100 trials per day and was conducted 5 days per week for 4 weeks, starting on the fourth day following PT. The control procedure for rats without rehabilitative training consisted of allowing the rats to eat 40 food pellets from the floor of the reaching chamber.

#### Staircase test

The staircase test was used to assess non-trained skilled forelimb motor performance [17]. The staircase apparatus (Kagawakikai, Takamatsu, Japan) consisted of a chamber with a central platform for the rat to climb onto and a set of eight steps on either side. Three sucrose pellets (45 mg; Bioserve, Flemington, NJ, USA) were placed in a well on each step. Rats were placed in the chamber for 10 min and the number of pellets consumed was recorded. For acquisition of the skill, rats received sequential training 5 days per week for 2 weeks. The results of the 3 days prior to PT were averaged and used for pre-stroke performance. A few rats use only their tongues to obtain food pellets, not their hands, and these rats could consume up to 6 pellets by improving their tongue use. Therefore, to properly evaluate hand function, we used only rats whose mean number of pellets consumed in pre-stroke testing was greater than 6 for each side.

#### Ladder walk test

The ladder walk test was used to assess precise limb placing and stepping. Rats were tested for their ability to walk on a horizontal ladder with irregular spacing (1–3 cm) between rungs following a previously described procedure [18]. Animals were trained 1 day prior to pre-stroke testing and tested 1 day prior to and 4 and 28 days following stroke. Animals were trained and tested three times per session, and the average of the results from the 3 trials was used for the motor performance score. Test trials were recorded on video and analyzed in a blind manner. The total number of steps and foot faults were counted for each limb, and the probability of foot fault (foot faults/total steps) was calculated. Both total misses and slips were defined as foot faults. Slips included deep slips that caused a fall as well as slight slips that did not result in a fall. The animals that could not cross the ladder in pre-stroke testing were excluded.

#### Cylinder test

The cylinder test was used to assess asymmetries in forelimb use for postural support during rearing [19]. Rats were placed individually in a clear plastic cylinder (height, 27 cm; diameter, 17 cm) for 5 minutes, and the number of times each forelimb was placed on the cylinder wall was counted. The test was performed 1 day prior to and 4 and 28 days following stroke. Performance was scored as the probability of impaired forelimb use (impaired forelimb use/total forelimb use). Animals that did not use either forelimb were excluded from the analysis.

### Photothrombosis

Photothrombotic stroke was induced as described previously [15] in the CFA contralateral to the dominant forelimb, as determined by the skilled forelimb reaching task. In the previous study, our stroke model destroyed approximately 90% of the CFA without any tissue damage in the Rosrtal forelimb area (RFA) [15]. Rats were anesthetized with an intraperitoneal injection of ketamine hydrochloride (60 mg/kg; Daiichi-Sankyou)/xylazine hydrochloride (6.0 mg/kg; Bayer, Leverkusen, Germany). To prepare the bone window, the skull over the CFA was carefully thinned (center: 3.5 mm lateral from bregma; radius: 2.0 mm) until the blood vessels of the brain surface could be clearly observed. For illumination, a green light source (532 nm; Nikon, Tokyo, Japan) attached to a 10× objective (Nikon) was used, resulting in a 4 mm diameter spot stereotaxically illuminated onto the bone window. We illuminated the brain for 15 minutes. During the first minutes of illumination, rose bengal (15 mg/kg; Wako Pure Chemical, Osaka, Japan) was injected via tail vein catheter (Terumo, Tokyo, Japan). Following PT, the catheter was removed and the incision closed. After the surgical procedure, the rats were kept on heating pads until awake and then returned to their home cages.

### Retrograde labeling of the neurons projecting to the lower cervical cord

#### Fast blue injections

Rats were anesthetized and maintained under a constant rate infusion of ketamine hydrochloride (48 mg/kg/h, intravenously) via tail vein catheter using a syringe pump (KD Scientific, Holliston, MA, USA). After skin incision and muscle separation from the vertebrae, a laminectomy was performed at the cervical spinal level from C5 to C7. Rats were fixed to a stereotactic frame (Narishige, Tokyo, Japan) by holding the dorsal processes at C4 and T1. The dura was then carefully opened and folded aside. Fast blue (1.25% in distilled water, Polysciences, Inc. Warrington, PA, USA) was injected into the spinal cord using a 34G beveled needle (NanoFil needle; World Precision Instruments, Sarasota, FL, USA) and a 10 μl syringe driven by an electric pump with a flow rate of 200 nl/min. Five injections of Fast blue (80 nl/injection) were made in the contralesional side of the cervical spinal cord (spinal level of C7 to C8: Fig 1B). The first injection was made at the C7 level with the needle tip positioned at 0.6 mm lateral to the midline at a depth of 1.4 mm. Two minutes after the injection, the needle was retracted and moved 1 mm caudally for the next injection. This procedure was repeated five times. Immediately following surgery, rats received intraperitoneal injections of an analgesic (Rimadyl: 2.5 mg/kg; Pfizer, New York, NY, USA); this was repeated once daily for 3 days following the operation.

#### Tissue preparation

One week following tracer injection, rats were deeply anesthetized with pentobarbital (240 mg/kg body weight, intraperitoneal; Kyoritsu Pharmaceutical, Tokyo, Japan) and transcardially perfused with 200 ml of 0.01 M phosphate-buffered saline, followed by 200 ml of 4% phosphate-buffered paraformaldehyde. After cryoprotection with 10%, 20%, and 30% sucrose, brains were embedded in Tissue-Tec (O.C.T. Compound; Sakura, Tokyo, Japan) and rapidly frozen in a dry ice/ethanol bath. Brain and spinal cord tissues were cut into 20 μm thick coronal sections on a cryostat. Brain sections were prepared in 500 μm intervals from the rostral-most tip to the caudal end of medulla oblongata. The spinal cord sections were prepared at the C7 and C8 levels for injection validation. Frozen sections were mounted on hydrophilic glass (MAS coat; Matsunami, Osaka, Japan) and coverslipped with Fluoromount (Diagnostic BioSystems, Pleasanton, CA, USA).

#### Injection validation

Images of the spinal cord sections at the C7 and C8 levels were taken using an all-in-one fluorescence microscope (4× objective: BZ-X700; Keyence, Osaka, Japan) and leakage of the tracer was carefully checked. Rats were excluded if leakage of the tracer was observed in the ipsilesional side of the spinal cord or in any part of the white matter. To confirm equivalent injections in the two groups, the diffusion of the tracer was examined by measuring the areas with increased background signals (intensity threshold 180–255) using imageJ software (NIH, Bethesda, MD, USA). The coordinates of the tracer diffusion centroid with the origin located at the center of the central canal were also measured.

#### Quantification of Fast blue-positive cells

Images from 39 sections at 500 μm intervals were taken using an all-in-one fluorescence microscope (10× objective: BZ-X700; Keyence, Osaka, Japan). Images were tiled and stitched using automatic software (BZ-X Analyzer; Keyence). Images were analyzed with MetaXpress Imaging Analysis V2.0 (Molecular Devices, Sunnyvale, CA, USA) to count the number of Fast blue-positive cells. The number of cells was multiplied by the ratio of section thickness and the number of intervals, allowing for an estimation of the number of cells in the whole brain.

### Statistics

All data are reported as mean ± standard deviation (SD). Unpaired two-tailed t-tests were performed to compare the injection diffusion, the number of Fast blue-positive cells in each brain area and behavioral measurements in the final session of the skilled forelimb reaching test. We compared behavioral performance in multiple groups at different time points using two-way repeated measures analysis of variance (ANOVA). For post-hoc testing, Tukey’s multiple comparison tests were used to compare the differences between the different time points and Sidak’s multiple comparison tests were used to detect group differences at each time point. A statistically significant difference was defined as a P value less than 0.05. Nonsignificant tendencies were indicated as (not significant but p < 0.1). All statistical analyses were carried out using GraphPad Prism Version 7.0 (GraphPad Software Inc. La Jolla, CA, USA).

## Results

### Number of animals analyzed and excluded

In this study, a total of 44 rats were used. Following the behavioral procedures, a total of 13 rats were excluded from the study because of a pre-infarct motor performance less than 30% (n = 11), or a post-infarct motor performance greater than 50% (n = 2). Thus, 31 rats were included in this study (PT: n = 15; PT + Rehab: n = 16). For some rats, we could not obtain usable data for statistical analysis from the cylinder test (no touches to the wall: n = 5), ladder walk test (no attempt to cross the ladder: n = 2), or staircase test (no hand use, or fewer than 6 pellets consumed in pre-infarct testing: n = 4). In the Fast blue tracer studies, nine rats were excluded because tracer leakages were observed in the ipsilesional spinal cord or the white matter.

### Rehabilitative skilled forelimb training improves functional recovery in a task-specific manner

To investigate the effect of rehabilitative skilled forelimb training on functional recovery following stroke, motor performance was assessed using various motor tests. Cylinder tests and ladder walk tests were conducted for the evaluation of general motor performance. These tasks can be executed with little habituation (3 trials for the ladder walk test) or without any training (cylinder test). The skilled forelimb test and staircase test were used to assess skilled motor performance. Performance of these tests requires a substantial training period (greater than 2 weeks).

**Fig 2:**
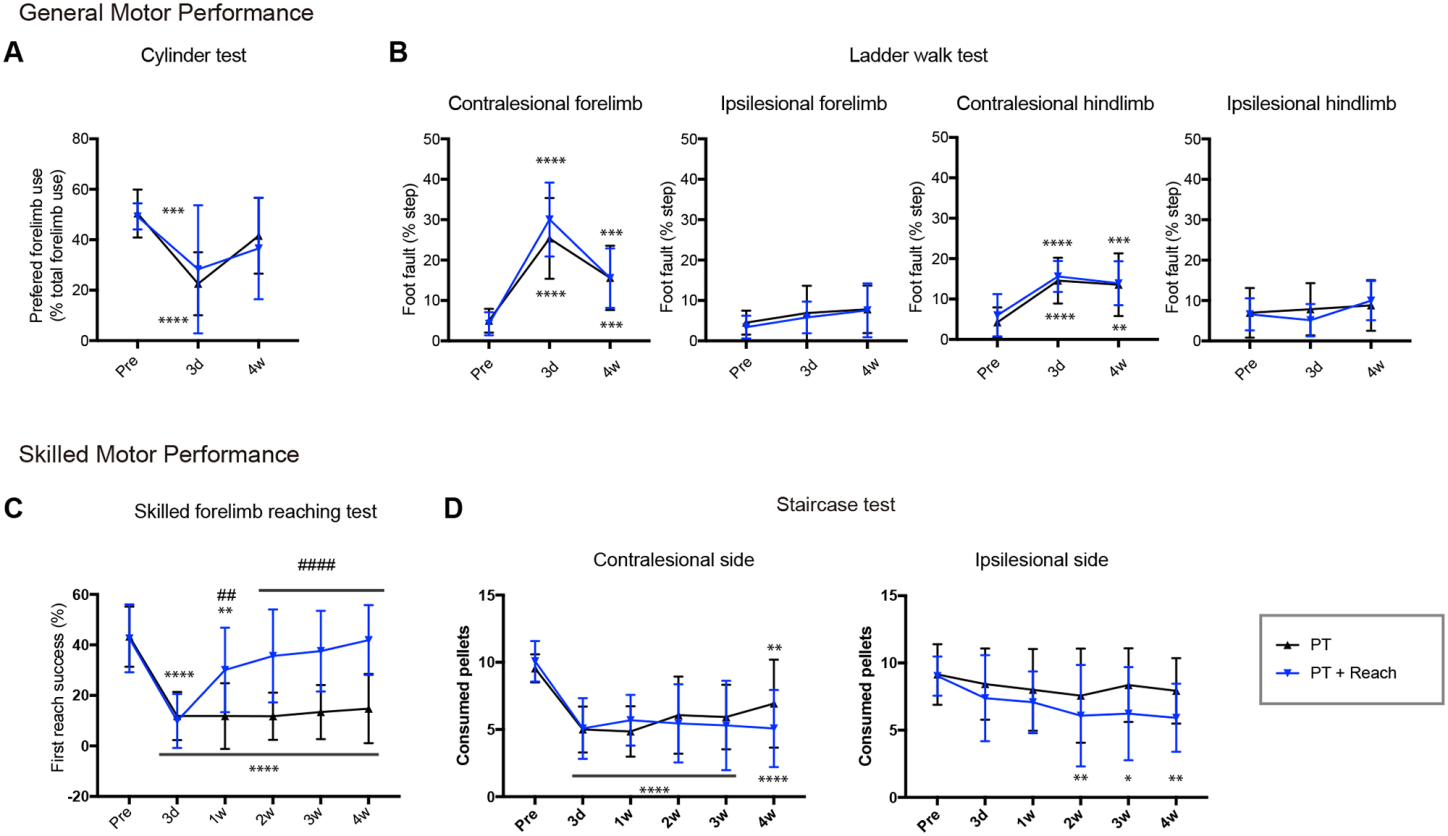
The effect of skilled forelimb training on functional recovery following stroke. (A) Results of the cylinder test. Rats showed a significant decrease in use of the preferred forelimb following stroke. Preferred forelimb use recovered in both groups. (B) Results of the ladder walk test. Stroke increased foot fault in the contralesional forelimb and hindlimb. Although the amount of foot fault decreased slightly in the contralesional forelimb 4 weeks following stroke, sustained disabilities were observed both in the contralesional forelimb and hindlimb. (C) Results of the skilled forelimb reaching test. Stroke caused severe disability in the skilled forelimb reaching task. While rats without rehabilitative training showed almost no improvement in motor performance, rats with rehabilitative training achieved nearly full recovery. (D) Results of the staircase test. Stroke caused impaired motor performance in the contralesional side. The impairment persisted until 4 weeks following stroke in both the untrained and trained rats. Unexpectedly, the trained rats showed a significant decline in motor performance in the ipsilesional (less affected) side. Repeated measures two-way ANOVA, followed by post-hoc test: Tukey’s multiple comparison test for the comparisons between the different time points. *p<0.05, **p<0.01, ***p<0.001, ****p<0.0001 vs. pre-stroke in the same group, Sidak’s multiple comparison test for the comparison between groups at each time point. ##p<0.01, ####p<0.0001 PT vs. PT + Rehab.

In the cylinder test (Fig 2A; PT: n = 12; PT + Rehab: n = 14), PT significantly decreased contralesional forelimb use at day 3 following PT (PT pre: 50.4 ± 9.5%; PT 3d: 22.5 ± 12.4%; P < 0.0001: Tukey’s test). Decreased contralesional forelimb use was recovered 4 weeks following PT (PT pre: 50.4 ± 9.5%; PT 4w: 41.5 ± 14.9%; P = 0.2918: Tukey’s test). No significant difference was observed between the PT and PT + Rehab groups (effect of group: F (1, 24) = 0.0008; P = 0.9773: Two-way repeated measures ANOVA). In the ladder walk test (Fig 2B; PT: n = 14; PT + Rehab: n = 15), PT significantly increased foot fault in the contralesional forelimb (PT pre: 5.0 ± 2.9%; PT 3d: 25.3 ± 9.9%; P < 0.0001: Tukey’s test) and hindlimb (PT pre: 4.2 ± 3.7%; PT 3d: 14.5 ± 5.6%; P < 0.0001: Tukey’s test), while no impairments were observed in the ipsilesional forelimb (PT pre: 4.5 ± 3.0%; PT 3d: 6.8 ± 6.7%; P = 0.390: Tukey’s test) or hindlimb (PT pre: 6.9 ± 6.1%; PT 3d: 7.8 ± 6.4%; P = 0.881: Tukey’s test). Although contralesional impairment persisted for 4 weeks following PT in both the forelimb (PT pre: 5.0 ± 2.9%; PT 4w: 15.5 ± 7.9%; P < 0.0008: Tukey’s test) and hindlimb (PT pre: 4.2 ± 3.7%; PT 4w: 13.5 ± 7.8%; P < 0.0002: Tukey’s test), rehabilitative training did not lead to significant improvement (effect of group, contralesional forelimb: F (1, 27) = 0.6647; P = 0.422; contralesional hindlimb: F (1, 27) = 1.032; P = 0.318; two-way repeated measures ANOVA). Skilled motor performance with the contralesional forelimb was severely impaired both in the skilled forelimb reaching test (Fig 2C; PT: n = 15; PT + Rehab: n = 16; PT pre: 43.2 ± 11.9%; PT 3d: 11.8 ± 9.5%; P < 0.0001: Tukey’s test) and in the staircase test (Fig 2D; PT: n = 14; PT + Rehab: n = 13; PT pre: 9.5 ± 1.0 PT 3d: 5.0 ± 1.9; P < 0.0001: Tukey’s test). These impairments persisted for 4 weeks following PT. Rehabilitative training significantly improved functional recovery in the skilled forelimb reaching task starting from 1 week following PT (Fig 2C; PT 4w: 14.7 ± 13.7%; PT + rehab 4w: 41.9 ± 13.9%; P < 0.0001: Sidak’s test), and first reach success rate recovered completely (PT + rehab 4w: 102.4 ± 33.9% of pre-stroke value). Skilled forelimb reaching training also significantly increased the number of total successes (= the number of pellets eaten in a session; PT: 8.9 ± 8.4; PT + rehab: 21.7 ± 7.0; P < 0.0001: Unpaired t-test), total success rate (= total successes / trial; PT: 20.0 ± 16.1%; PT + rehab: 43.8 ± 14.3%; P = 0.0001: Unpaired t-test) and percentage of successful reaches (total successes / total reaches; PT: 16.7 ± 15.0%; PT + rehab: 42.5 ± 13.5%; P < 0.0001: Unpaired t-test) 4 weeks after stroke. On the other hand, rehabilitative training did not affect functional recovery for the staircase test (PT 4w: 6.9 ± 3.3; PT + rehab 4w: 5.07 ± 2.9; effect of group: F (1, 25) = 0.173; P = 0.2255; two-way repeated measures ANOVA). Furthermore, rats with rehabilitative training showed a slight decline in motor performance in the staircase test of the ipsilesional forelimb (PT + Rehab pre: 9.0 ± 1.5; PT + rehab 4w: 5.9 ± 2.5; P = 0.0039; Tukey’s test).

### Validation of Fast blue injections

**Fig 3:**
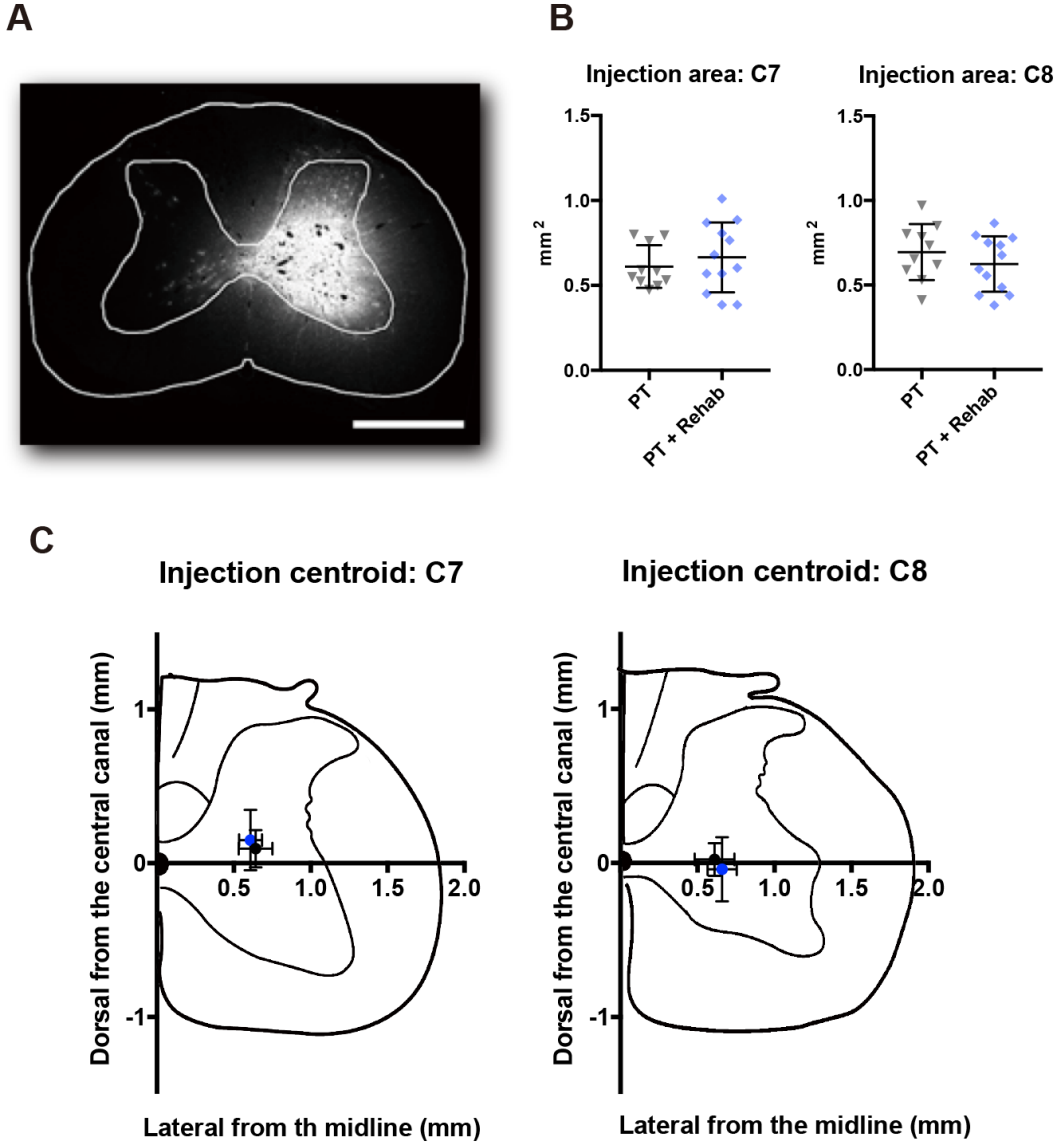
Tracer injection validation. (A) Representative photo of the Fast blue injection in the spinal cord. Scale bar = 1 mm. (B) Injection area of Fast blue at the C7 and C8 spinal levels was not significantly different between groups. (C) There were no significant differences between groups in the location of the injection centroid at the C7 and C8 spinal levels.

Following a 4-week recovery period, all rats were injected with Fast blue in the contralesional lower cervical spinal cord (C7-8). The injection site was carefully checked and rats were excluded if leakage of the Fast blue injection was observed in any part of the white matter or on the contralateral side of the injection (PT: n = 5; PT + Rehab: n = 4). Because each descending spinal pathway enters the gray matter of the spinal cord from different parts of the white matter, both the volume and location of the injection can affect the number of the Fast blue labeled cells in each brain area. Thus, we confirmed whether an equivalent volume of tracer was injected into the same area of the spinal cord for each group (Fig 3; PT: n = 10; PT + Rehab: n = 12). The Fast blue injection area was not significantly different at either the C7 (PT: 0.61 ± 0.13 mm^2^; PT + Rehab: 0.71 ± 0.19 mm^2^; P = 0.4755; unpaired t-test) or C8 level (PT: 0.69 ± 0.17 mm^2^; PT + Rehab: 0.65 ± 0.16 mm^2^; P = 0.3328; unpaired t-test). Similarly, the lateral and dorsal coordinates of the injection centroid were not significantly different between the groups at either the C7 (lateral, dorsal from the central canal; PT: 0.64 ± 0.11 mm, 0.09 ± 0.12 mm; PT + Rehab: 0.60 ± 0.07 mm, 0.15 ± 0.20 mm; P = 0.3095, P = 0.3031) or C8 level (lateral, dorsal from the central canal; PT: 0.61 ± 0.13 mm, 0.02 ± 0.11 mm; PT + Rehab: 0.66 ± 0.09 mm, -0.04 ± 0.21 mm; P = 0.4179, P = 0.3147).

### Rehabilitative skilled forelimb training increases the number of neurons projecting to the lower cervical spinal cord from the ipsilesional cerebral cortex

**Fig 4:**
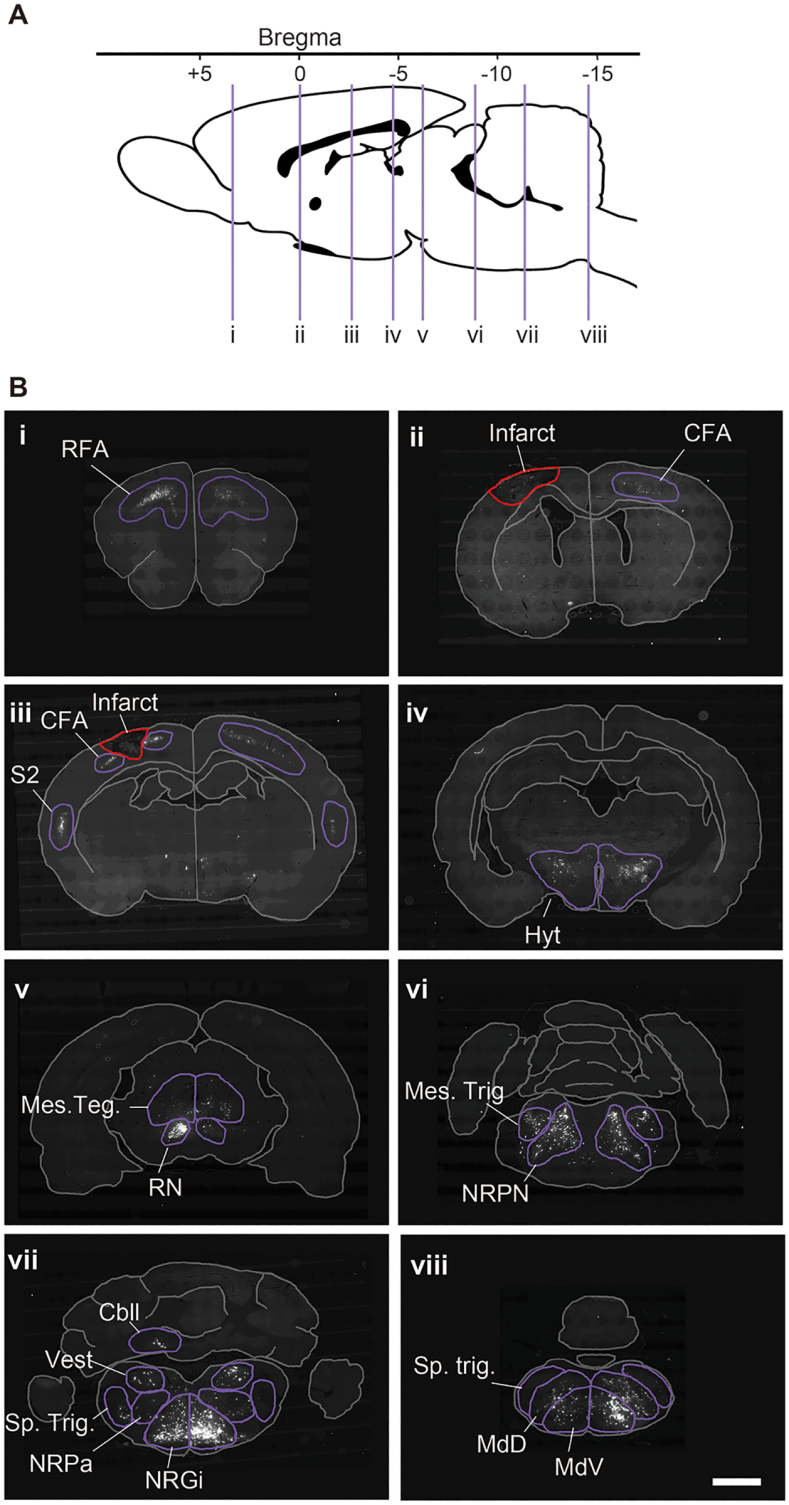
Distribution of Fast blue-positive neurons. (A) The sagittal brain atlas indicating the level of the section in B (purple line; i-viii). (B) Representative pictures of the brain sections in the cerebral cortex (i, ii and iii), thalamus and hypothalamus (iv), midbrain (v), pons (vi) and medulla oblongata (vii and viii). The neurons projecting to the contralesional spinal cord at C7-8 were labeled with Fast blue. Infarct is indicated with a red line and the observed area of Fast blue-positive cells are indicated with a purple line. The names of the brain areas are abbreviated as follows: Rostral forelimb area (RFA), caudal forelimb area (CFA), secondary sensory area (S2), hypothalamic area (Hyt), mesencephalic tegmental area (Mes. Teg.), red nucleus (RN), nucleus reticularis pontis (NRPN) mesencephalic trigeminal nuclei (Mes. Trig.), deep cerebellar nuclei (Cbll), nucleus reticularis gigantocellularis (NRGi), nucleus reticularis parvocellularis (NRPa), spinal trigeminal nuclei (Sp. Trig.), vestibular nuclei (Vest), dorsal medullary reticular nucleus (MdD), and ventral medullary reticular nucleus (MdV). Scale bar = 1 mm.

**Fig 5:**
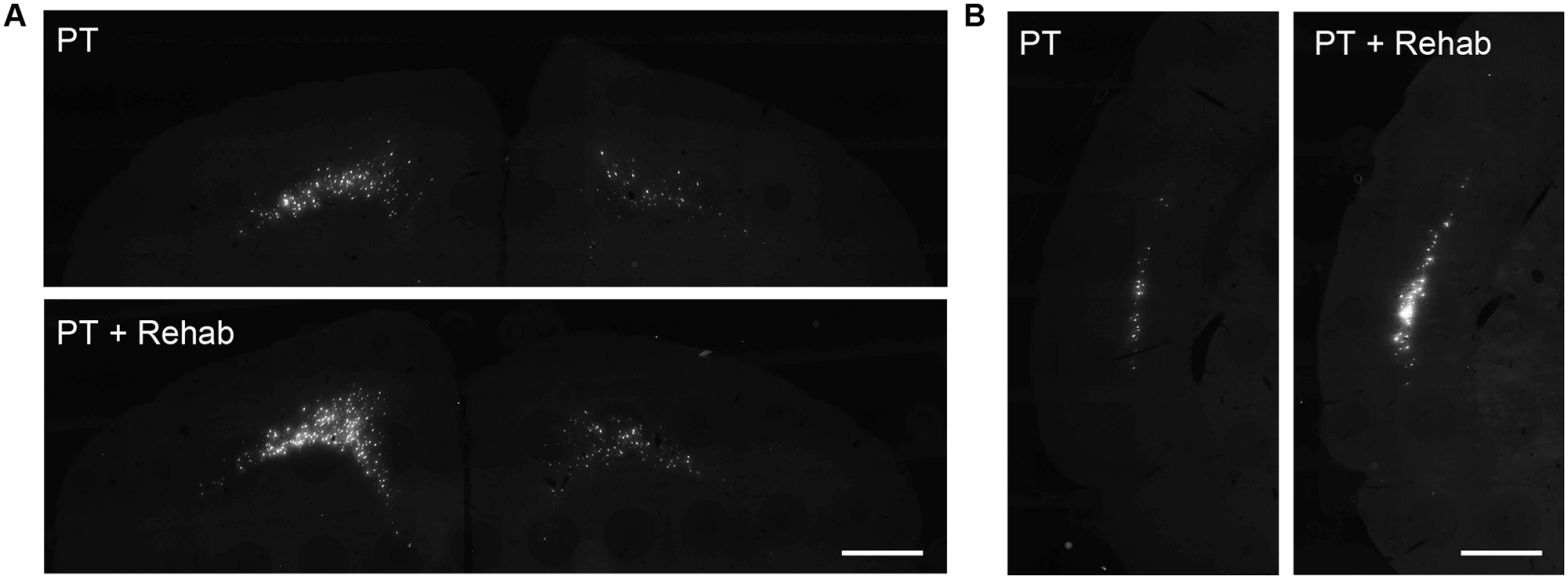
Distribution of Fast blue-positive neurons in the cerebral cortex. Representative pictures of Fast blue labeled brain sections in the rostral forelimb area (A) and secondary sensory area (B) of the PT rats and the PT + Rehab rats. The ipsilesional hemisphere is located on the left side. Scale bars = 1 mm.

**Fig 6:**
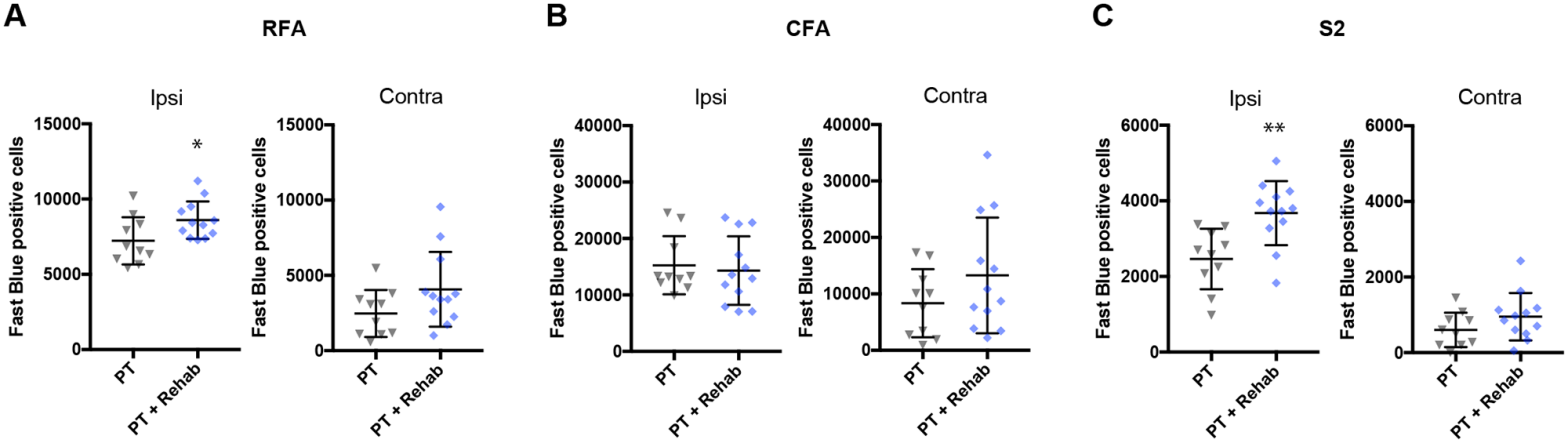
The effect of skilled forelimb training on the spinal projections from the cerebral cortex. The numbers of Fast blue-positive cells were analyzed in the ipsilesional (Ipsi) and contralesional (Contra) rostral forelimb area (A: RFA), and in the caudal forelimb area (B: CFA) and secondary sensory area (C: S2). Rehabilitative skilled forelimb training increased the number of Fast blue-positive cells in the ipsilesional RFA and the S2. Unpaired t-test: *p<0.05, **p<0.01 PT vs. PT + Rehab.

Brain sections were prepared in 500 μm intervals from the rostral-most tip to the end of the medulla oblongata. Fast blue-positive cells were identified automatically by image software, and counted in each brain area according to their location on a brain atlas (Fig 4A; PT: n = 10; PT + Rehab: n = 12). The photothrombotic stroke induced an infarct located in the CFA (Fig 4Bii) without visible histological damage in the RFA (Fig 4Bi). Although a major portion of the CFA was destroyed by the stroke, some Fast blue-positive cells remained in the caudal part of the CFA (Fig 4Biii). In the ipsilesional cortex, significant increases in Fast blue-positive cells were detected in the RFA (Figs 5A and 6A; PT: 7225 ± 1569; PT + Rehab: 8600 ± 1248; P = 0.033; unpaired t-test) and secondary sensory cortex (S2) (Figs 5B and 6C; PT: 2463 ± 799; PT + Rehab: 3675 ± 848; P = 0.0027; unpaired t-test), while rehabilitative training did not alter the remaining Fast blue-positive cells in the ipsilesional CFA (Fig 6B; PT: 15263 ± 5138; PT + Rehab: 14331 ± 6064; P = 0.7052; unpaired t-test). Although rehabilitative training also slightly increased the number of Fast blue-positive cells in the contralesional cortex, no significant difference was observed between groups (Fig 6A–6C; RFA; PT: 2468 ± 1558; PT + Rehab: 4073 ± 2485; P = 0.092; unpaired t-test, CFA; PT: 8343 ± 6032; PT + Rehab: 13269 ± 10258; P = 0.1968; unpaired t-test, S2; PT: 602 ± 457; PT + Rehab: 950 ± 627; P = 0.160; unpaired t-test).

### Rehabilitative skilled forelimb training does not significantly alter the number of neurons projecting to the lower cervical spinal cord from any brain areas in the hypothalamus, midbrain, pons, or medulla oblongata

**Fig 7:**
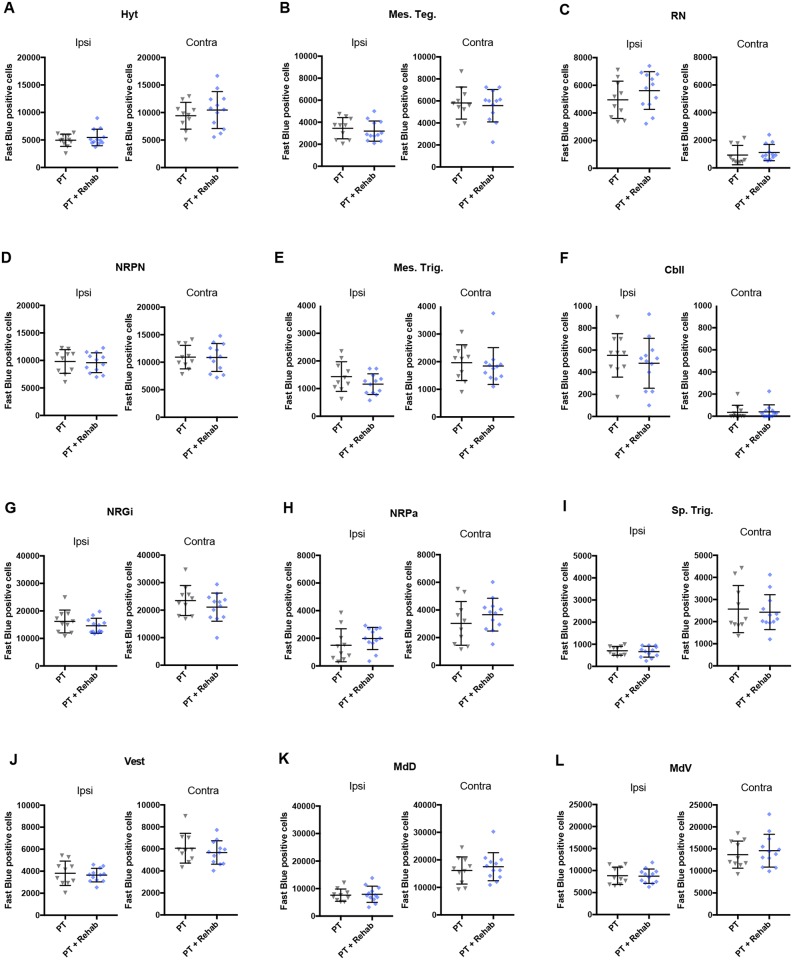
The effect of skilled forelimb training on the spinal projections from the brainstem. The numbers of Fast blue-positive cells were analyzed in the (A) Hypothalamic area (Hyt), (B) mesencephalic tegmental area (Mes. Teg.), (C) red nucleus (RN), (D) nucleus reticularis pontis (NRPN) (E) mesencephalic trigeminal nuclei (Mes. Trig.), (F) deep cerebellar nuclei (Cbll), (G) nucleus reticularis gigantocellularis (NRGi), (H) nucleus reticularis parvocellularis (NRPN), (I) spinal trigeminal nuclei (Sp. Trig.), (J) vestibular nuclei (Vest), (K) dorsal medullary reticular nucleus (MdD), and (L) ventral medullary reticular nucleus (MdV). Rehabilitative skilled forelimb training did not induce significant changes in any areas of the hypothalamus, midbrain, pons, cerebellum, or medulla oblongata.

To investigate the effect of rehabilitative skilled forelimb training on axonal remodeling in the descending spinal pathway from the deep brain areas, distribution of the spinal projecting neurons in the deep brain areas were examined. As reported in a previous study [10], while contralateral projections were dominant in the corticospinal and rubrospinal pathways, hypothalamus and brainstem-spinal projections showed ipsilateral dominance (Figs 4 and 7). The Fast blue-positive cells were counted in the hypothalamus (Fig 7A: hypothalamic area), mid brain (Fig 7B: mesencephalic tegmental area; Fig 7C: red nucleus), pons (Fig 7D: nucleus reticularis pontis; Fig 7E mesencephalic trigeminal nuclei), cerebellum (Fig 7F: deep cerebellar nuclei), medulla oblongata (Fig 7G: nucleus reticularis gigantocellularis; Fig 7H: nucleus reticularis parvocellularis; Fig 7I: spinal trigeminal nuclei; Fig 7J: vestibular nuclei; Fig 7K: dorsal medullary reticular nucleus; Fig 7L: ventral medullary reticular nucleus). In contrast to the cerebral cortex, rehabilitative training did not induce significant changes in the number of Fast blue-positive cells in any of these brain areas in either the ipsilesional or contralesional hemisphere (Fig 7A–7L). We did not observe even a non-significant tendency in these areas (Table 1).

**Table 1:** Summary of the histological changes induced by rehabilitative skilled forelimb training.

Ipsilesional				Contralesional			
	Difference	P value	Significance		Difference	P value	Significance
RFA	1375 ± 496	0.0330	*	RFA	1605 ± 907	0.0920	~
CFA	-931 ± 2426	0.7052	n.s.	CFA	4926 ± 3690	0.1968	n.s.
S2	1213 ± 354	0.0027	**	S2	347 ± 238	0.1606	n.s.
Hyt	504 ± 553	0.3744	n.s.	Hyt	1028 ± 1272	0.4282	n.s.
Mes. Teg.	-261 ± 401	0.5229	n.s.	Mes. Teg.	-1550 ± 1063	0.7018	n.s.
RN	666 ± 580	0.2647	n.s.	RN	184 ± 275	0.5103	n.s.
NRPN	-226 ± 839	0.7900	n.s.	NRPN	-71 ± 1011	0.9442	n.s.
Mes. Trig	-272 ± 194	0.1769	n.s.	Mes. Trig	-121 ± 281	0.6710	n.s.
Cbll	-71 ± 91	0.4440	n.s.	Cbll	4 ± 27	0.8680	n.s.
NRGi	-1555 ± 1475	0.3043	n.s.	NRGi	-2384 ± 2259	0.3039	n.s.
NRPa	487 ± 426	0.2664	n.s.	NRPa	630 ± 589	0.2978	n.s.
Sp. Trig.	-242 ± 162	0.6816	n.s.	Sp. Trig.	-138 ± 395	0.7303	n.s.
Vest	-168 ± 372	0.6556	n.s.	Vest	-389 ± 513	0.4568	n.s.
MdD	345 ± 1128	0.7626	n.s.	MdD	1347 ± 2150	0.5380	n.s.
MdV	-107 ± 768	0.8960	n.s.	MdV	902 ± 1478	0.5485	n.s.

Unpaired t test: PT vs. PT + Rehab. *p<0.05, **p<0.01, ~Not significant (but p < 0.1), n.s. not significant (p > 0.1).

## Discussion

### The effect of rehabilitative skilled forelimb training on functional recovery

Consistent with a previous study [15], photothrombotic stroke in the CFA caused severe disability in both general and skilled motor tasks. Untrained rats showed almost no recovery in skilled motor performance. In contrast, rehabilitative skilled forelimb training significantly improved motor performance as assessed by first reach success rate in the skilled forelimb reaching task, while rehabilitative training did not show any significant improvement in the other tasks. Skilled forelimb training also increased other measures such as total successes, total success rate, and percentage of successful reaches in the final testing session of the skilled forelimb reaching test. These results indicate that skilled forelimb training improves the accuracy of pellet retrieval movements. Although both functional recovery and compensation contribute to improved motor performance after stroke [20], a previous study has shown [15] that rehabilitative training with a skilled forelimb reaching task normalizes skilled reach movements. Thus, it is thought that the skilled forelimb training induced task-specific recovery but not compensation. The translatability of the functional recovery induced by the skilled forelimb task differs between experiment settings. For example, Starkey et al. reported that skilled forelimb reach training (50 successful attempts, 5 days per week for 6 weeks) after unilateral pyramidotomy led to an improved success rate in the horizontal ladder walk and the staircase task [21], while Nakagawa et al. reported that skilled forelimb reach training (60 attempts, 6 days per week for 4 weeks) after traumatic brain injury improved the success rate only in the skilled forelimb reaching test but not in the staircase test, ladder walk test, or Capellini handling test [22]. These differences may be a result of the intensity of the training or the severity and type of injury. Previous repetitive transcranial magnetic stimulation studies in stroke patients suggested a bimodal balance-recovery model, in which activity in the residual network causes vicariation or intra- and inter-hemispheric inhibition, depending on the severity of the injury [23]. Vicariation, or substitution of lost function by the residual network, would dominate in patients with more severe damage [24], suggesting a greater restorative effect of neural network remodeling in more severely affected individuals. Interestingly, general functional improvement was induced by skilled forelimb reach training only when training enhanced axonal remodeling in the corticospinal tract from the contralesional hemisphere; Starkey et al. [21] reported enhanced axonal remodeling but we [15] and Nakagawa et al. [22] did not observe a significant effect in the corticospinal tract from the contralesional hemisphere. Therefore, the extent of the brain area in which training induces axonal plasticity may be a deciding factor in the translatability of rehabilitation-induced functional recovery.

During the recovery period following stroke, we unexpectedly found that rehabilitative training slightly worsened ipsilesional (less affected) forelimb function in the staircase test. Several studies have reported impaired ipsilesional motor performance in stroke patients [25, 26]. These impairments are likely caused by the destruction of ipsilateral corticospinal projections. However, in this study, ipsilesional forelimb function was not impaired on day 3 following stroke in either the ladder walk test or the staircase test. Therefore, ipsilesional forelimb dysfunction seems to be associated with the recovery process. Maladaptive effects of rehabilitative training on trained forelimb function have been demonstrated in several studies. In rats with spinal cord injury, skilled forelimb training improved motor behavior in trained tasks, but increased missteps in the ladder walk task [27]. Skilled forelimb training with the ipsilesional (less affected) forelimb also impedes functional recovery in the contralesional forelimb [28]. As for the mechanisms of maladaptive effects of rehabilitative training, the competition of neural substrates and maladaptive neural network remodeling for untrained tasks may be involved. These mechanisms might also cause the maladaptive effects of rehabilitative training on ipsilesional forelimb function.

### The effects of skilled forelimb training on axonal remodeling in the corticospinal pathway

Since each descending spinal pathway passes through a different part of the spinal white matter and has different terminal distribution patterns in the spinal grey matter [29], both the volume and the location of the tracer injection affect the distribution of the labeled neurons in the brain. Thus, we validated the extent and localization of the tracer in all rats. We confirmed equivalent injection diffusion and similar localization of the centroid of the tracer diffusion between groups, indicating that the group difference in the histological analysis must be caused by the rehabilitative treatment rather than the tracer injection. In the analysis of the cerebral cortex, we found that rehabilitative training increased the number of Fast blue-positive cells in the ipsilesional RFA, consistent with a previous study [15]. In addition to the RFA, the current study revealed an increased number of Fast blue-positive cells in the S2. This change was more prominent than the change in the RFA. The axonal remodeling in the S2 has been observed in spontaneous recovery after stroke [10] and can be promoted by anti-NogoA antibody therapy [6]. Some human studies have also reported changes in neural activity in the S2 after stroke. Johansen-Berg et al. reported that therapy‐related improvements in hand function are correlated with increases in fMRI activity in the ipsilesional premotor cortex and the S2 [30]. Dobkin et al. reported that activation in the bilateral S2 increased over the time with functional gain [31]. These data imply a possible contribution of the S2 in functional recovery after stroke. However, our previous study revealed that if the remaining RFA is destroyed after completion of rehabilitative training, recovered function is completely abolished despite preservation of the S2 [15]. Therefore, the RFA plays a central role in rehabilitation-induced task-specific recovery and the S2 may have only a supportive role, such as for the processing of somatosensory perception [32] and perceptual learning [33]. In contrast to the ipsilesional cortex, skilled forelimb training did not increase the number of Fast blue-positive cells in the contralesional cortex, indicating that axonal remodeling is not induced in the corticospinal pathway from the contralesional cortex. Because the area where neural network remodeling occurs is influenced by the severity of injury and by regional neural activity, axonal remodeling in the contralesional cortex should also be affected by these factors, as is the case for axonal remodeling in the brainstem-spinal pathway, as described below.

### The effects of skilled forelimb training on axonal remodeling in the brainstem-spinal pathways

In the analysis of the brainstem, we did not observe a significant difference in the number of Fast blue-positive cells in any areas. We analyzed 26 areas in the brainstem and did not find even a weak tendency towards change (p > 0.1 in all areas). These results indicate that rehabilitative skilled forelimb training promotes axonal remodeling in the corticospinal pathway but not in the brainstem-spinal pathways. Axonal remodeling in the brainstem-spinal pathways has been reported in spontaneous recovery after spinal cord injury [34] and stroke [10], and axonal remodeling in these pathways can be promoted by combined treatment with chondroitinase ABC injection and rehabilitative training [35], as well as by genetic ablation of the Nogo receptor 1 [34], indicating intrinsic axonal plasticity in brainstem-spinal pathways. A possible reason as to why skilled forelimb training did not promote axonal remodeling in the brainstem-spinal pathways is that the stroke model used in the current study might be too mild to induce neural network remodeling in deep brain areas. Previous studies have demonstrated that the extent of the area that undergoes neural network remodeling depends on the degree of brain damage. For example, greater destruction of the primary motor cortex results in more prominent motor map reorganization in the remote motor cortex [36, 37]. Furthermore, while rats subjected to middle cerebral artery occlusion (MCAO), which destroys both cortical and subcortical regions and causes axonal sprouting of corticospinal tract from the contralesional motor cortex [38], MCAO rats with only subcortical lesions do not undergo remodeling of the corticospinal tract [39]. In fact, spontaneous axonal remodeling in the brainstem-spinal pathways has been observed in large stroke models (less than 10% of ipsilesional corticospinal neurons spared [10]) and severe spinal cord injury (complete hemisection [40]). Compared with these injury models, our stroke model spares a larger number of intact corticospinal neurons (34.4% of ipsilesional corticospinal neurons spared [15]). Therefore, it is likely that the severity of brain damage affects the extent of the area where rehabilitative training promotes axonal remodeling. In addition to the degree of brain damage, the intensity and type of training may also influence the area where rehabilitative training induces axonal remodeling. Neural plasticity after brain injury is activity dependent [41] and the level of brain-derived neurotrophic factor increases depending on rehabilitation intensity [42]. We determined the intensity of training based on the behavioral improvement and motor map reorganization induced by post-stroke training. In a previous study, we observed significant enlargement of the RFA as determined by intracortical microstimulation, and task specific recovery as evaluated using the skilled forelimb reaching test, in rats trained in the skilled forelimb reaching task for 50 trials/day, 5 days/week for 4 weeks [15]. This intensity of training also caused an increase in corticospinal axon fiber density in the lower spinal cord, which controls distal forelimb movement [15]. In the present study, we doubled the amount of training, to create clearer differences for observation (100 trials/day, 5 days/week for 4 weeks). This level of training is more intense than was used in a protocol that resulted in significant motor map reorganization (50 trials/day, 5 days/week for 5 weeks) [13], or in axonal remodeling in the corticorubral pathway (40 trials/session, 18 training sessions starting 6 days after lesion and ending at 29 days after lesion) [5]. Thus, our training intensity should be enough to induce cortical plasticity. Since different forms of motor tasks induce distinct patterns of regional brain activation [43], different training methods, or more intense training, might induce axonal remodeling in the brainstem-spinal pathways by increasing neural activity in the brainstem. An optimal training protocol to induce axonal remodeling in both corticospinal and brainstem-spinal pathway might also facilitate improvement of both general and skilled motor performance, because corticospinal and brainstem-spinal pathways are associated with execution of precise skilled movement and gross motor function, respectively. It is possible that a combination therapy comprising both a skilled task and a gross motor task, such as the rotarod, may be beneficial for this purpose.

In the current study, rehabilitative training was carried out for 4 weeks, beginning on day 4 after stroke induction. A great deal of evidence from animal [44] and clinical studies [45] indicates that this subacute period is a critical period for stroke recovery, in which the brain is most receptive to modification by rehabilitative experience, and delayed rehabilitative treatment is less effective than when initiated at earlier time points. These data suggest that neural network remodeling induced by skilled forelimb training should be less prominent if the training is initiated in the chronic phase of a stroke. However, Stinear et al. demonstrated that functional improvement in chronic stroke patients (at least 6 months after stroke) declines with increasing corticospinal tract disruption [46], suggesting that plasticity of the corticospinal pathway remains critical during the chronic phase of stroke recovery. Since it has been suggested that upregulation of growth-inhibiting genes limits the efficacy of rehabilitative training in the chronic phase [47], an important challenge will be to find ways to widen this therapeutic time window [48].

It should be pointed out that we cannot exclude the functional contribution of the brainstem-spinal pathways. Studies using retrograde tracers cannot rule out axonal remodeling and synapse formation in the neurons that originally had neural projections to the injection site. Moreover, rehabilitative training may modify the function of the brainstem-spinal pathways by increasing the neural input from the cerebrobulbar pathways [4, 5]. However, previous studies have reported that if the majority of the corticospinal neurons are destroyed, rehabilitative training alone does not lead to substantial recovery in skilled forelimb function [3, 15] despite intact brainstem-spinal pathways. These results are consistent with clinical reports that have shown that the functional potential in chronic stroke patients depends on the integrity of the ipsilesional corticospinal tract [46, 49]. Therefore, plasticity of the cortical neurons projecting to the spinal cord through either direct (corticospinal neuron) or indirect (corticobulbar neuron) pathways should be necessary for the recovery of skilled forelimb tasks induced by rehabilitative training.

In conclusion, rehabilitative skilled forelimb training induces task-specific functional recovery and promotes axonal remodeling selectively in the corticospinal pathway. Our results suggest that cortical plasticity, rather than brainstem plasticity, plays a dominant role in task-specific recovery induced by rehabilitative training after subtotal destruction of the motor cortex.
